# Epidemiology of the first outbreak of carbapenem-resistant *Klebsiella pneumoniae* in Saudi Arabia

**DOI:** 10.1186/1753-6561-5-S6-P295

**Published:** 2011-06-29

**Authors:** HH Balkhy, A El-Saed, S Al Johani, HT Tayeb, A Al-Qahtani, M Alahdal, M Sallah, A Alothman, Y Alarabi

**Affiliations:** 1 Infection Prevention and Control Department, KAMC, Riyadh, Saudi Arabia; 2 Molecular Department, King Abdullah Internatinal Medical Research Center, Riyadh, Saudi Arabia; 3 Clinical Pathology Department, KAMC, Riyadh, Saudi Arabia; 4 King Faisal Specialist Hospital and Research Center, Riyadh, Saudi Arabia; 5 Department of Medicine, KAMC, Riyadh, Saudi Arabia; 6 Department of Critical Care, KAMC, Riyadh, Saudi Arabia

## Introduction / objectives

We describe our experience in detecting and containing the first documented CRKP outbreak in Saudi Arabia (SA).

## Methods

A prospective investigation of all cases identified with carbapenem resistance during the outbreak peroid and six months prior.

## Results

During March 2010, a cluster of 6 patients with CRKP was detected. Patients with CRKP were placed under strict contact isolation. Admission and periodic active surveillance cultures showed a downward trend of CRKP clinical cases over the following months to zeros in July and August. All patients had prolonged hospital stay before CRKP detection and the majority had recent history of carbapenem use (75%), colonization/infection with other MDROs (75%), surgical procedures (80%), indwelling devices (83%), and mechanical ventilation (75%). About 33% of patients with CRKP had clinical infection and 58% died during the current hospitalization. PFGE results identified a dominant clone during the outbreak, Figure 1.

## Conclusion

Infection control practices are not enough to eliminate the emergence of resistant pathogens. More active interventional methods including an antimicrobial stewardship program would be essential to combat the emergence of multidrug resistant organisms.

## Disclosure of interest

None declared.

